# Next-Day Discharge Is Feasible in Robotic-Assisted Thoracic Surgery Anatomical Lung Resections Irrespective of Patient Characteristics

**DOI:** 10.3390/jcm14093198

**Published:** 2025-05-05

**Authors:** Ra’fat Tawalbeh, William Ansley, Obada Alqudah, Ahmad Asqalan, Hammad Hassan, Bartolmiej Szafron, Cristina Viola, Jakub Kadlec, Waldemar Bartosik, Vasileios Kouritas

**Affiliations:** Department of Thoracic Surgery, Norfolk and Norwich University Hospital, Colney Lane, Norwich NR4 7UY, Norfolk, UK; rafatawuk@gmail.com (R.T.); william.ansley@nnuh.nhs.uk (W.A.); obada.alqudah@nnuh.nhs.uk (O.A.); ahmad.asqalan@nnuh.nhs.uk (A.A.); hammad.hassan@nnuh.nhs.uk (H.H.); bartlomiej.szafron@nnuh.nhs.uk (B.S.); cristina.viola@nnuh.nhs.uk (C.V.); jakub.kadlec@nnuh.nhs.uk (J.K.); waldemar.bartosik@nnuh.nhs.uk (W.B.)

**Keywords:** robotic, lung resection, next day, discharge

## Abstract

**Background:** Next-day discharge post-robotic-assisted thoracic surgery (RATS) anatomical lung resections are shown to be achieved in young and fit patients. This study aims to compare next-day discharge RATS anatomical lung resection patients matched with patients who stayed longer. **Methods:** A retrospective analysis of patients who underwent RATS anatomical lung resection by a single surgeon was conducted. Based on the variables found to be different, two propensity-matched groups were created: a next-day discharge group and a group of patients with longer stays. **Results:** This study included 202 patients, 49 (24.3%) of whom were discharged the next day. The mean age was 68.3 ± 9.8 years, and 114 (56.4%) patients were females. Based on the variables found different, two matched groups with 46 patients for age, gender, performance score, American Society of Anesthesiologists score, number of co-morbidities and Forced Expiratory Volume in 1 sec were created. Re-admissions, complications, and death rates were similar, but next-day discharge patients had more sublobar resections (65.2% vs. 37%, *p* = 0.029), shorter procedures (132 vs. 179 min, *p* = 0.048), and morning theater slots (71.7% vs. 32.6, *p* = 0.018). These were shown to be independent predictors of next-day discharge. Major air leak issues also kept patients in the hospital longer (23.9% vs. 6.5%, *p* = 0.024). **Conclusions:** Next-day discharge following RATS anatomical lung resection appeared to have no increased risk of re-admissions or complications, irrespective of fitness, age, or other patient characteristics. Patients receiving short-duration surgery and morning sublobar resections without major air leak issues have higher chances of achieving next-day discharge.

## 1. Introduction

The evolution from open thoracotomy to video-assisted thoracoscopic surgery (VATS) has made quick discharges for patients undergoing anatomical lung resections feasible [1,2,3]. Next-day discharge became feasible post-VATS procedures in small percentages, i.e., 4–5%, but was associated with variable re-admission rates, which in some series was shown to be as high as 14.2% [1,2,3]. Despite this, re-admissions were shown to be no more frequent than those discharged following longer inpatient stays [4].

Robotic-assisted thoracic surgery (RATS) is being utilized more and more frequently in practice, accomplishing similar, if not better, clinical outcomes when compared to VATS or open lung resections [5,6].

In RATS anatomical lung resections, limited and small single-institution studies have shown that a next-day discharge is feasible and does not lead to increased re-admissions when compared with all other longer-staying patients [7,8]. However, complications that develop in patients with anatomical lung resections would, by definition, lead to a prolonged in-hospital stay [9]. Concurrently, a prolonged in-hospital stay is expected in less fit and older patients with more co-morbidities [1,2,3,7,8].

In an effort to truly investigate if next-day discharge post-RATS anatomical resection is safe, irrespective of age, fitness, co-morbidities, etc., we compared the outcomes of patients who were discharged by the next-day post-RATS anatomical lung resection with propensity-matched patients who stayed longer than 1 day post-surgery.

## 2. Materials and Methods

### 2.1. Study Design

All patients who underwent RATS anatomical lung resections by one surgeon (in order to avoid bias from surgical trauma and variability in practice that would be induced by different surgeons) within a period of time between January 2021 and August 2024 were retrospectively investigated. Wedge resections, complex lung resections, including chest wall resection, intrapericardial dissections, sleeves etc., or pneumonectomies were excluded from the study. Overall, 202 patients were divided into a next-day discharge group (which included patients who were discharged within 24 h post-surgery) or a longer-stay discharge group (which included patients who stayed longer than 24 h in the hospital). After an initial comparison of the variables between the 2 groups, a propensity match analysis was conducted in a 1:1 matching manner using the “nearest neighbour” technique based on the variables identified to be different in order to create 2 new matched groups, including 46 patients each. Multiple intraoperative and postoperative variables were investigated between the matched groups.

### 2.2. Patient Data

The data collected included the following: Non-identifiable demographic details (age, gender); the number and type of co-morbidities; the body mass index (BMI); lung function tests (LFTs), including Forced Expiratory Volume in 1 sec (FEV_1_ in %) and the Transfer Factor for lung Carbon Monoxide (TLCO in %); the American Society of Anesthesiologists score (ASA); performance score (PS), as per the Eastern Cooperative Oncology Group (ECOG); the type (primary lung cancer, metastasis or other) and pathological stage of the cancer (for primary lung cancer); the type of resection (sublobar anatomical lung resection or lobectomy); upstaging due to N status; the number and reason of re-admissions; the returns to theater and interventions needed postoperatively (e.g., reinsertion of chest drain, subcutaneous cuts for surgical emphysema, etc.); planned and unexpected admissions to the Critical Care Complex Unit (CCC); the duration of the procedure; am or pm case in theater; and the overall length of in-hospital stay (LOS), which included the LOS from the procedure and the LOS because of re-admissions (if any).

The morbidity captured included the overall morbidity (considered when 1 or more complications in the same patient occurred on many occasions, during an in-hospital stay or after discharge was/were captured) and also separately during surgery admission or post-discharge: respiratory (atelectasis/pneumonia: diagnosed with clinical and radiological features, i.e., fever > 38 °C, increased inflammatory markers, atelectasis/consolidation seen on imaging, empyema, etc.), cardiac (including arrhythmia, heart failure, myocardial infarction, etc.) and renal/urinary (including acute kidney injury with abnormal urea/creatinine tests, renal failure, insertion of urine catheter, etc.) morbidity. Air leak complications captured included prolonged air leak (PAL, perceived as an air leak > 5 days) and discharges with a flutter bag. Pain complications were captured when patients had to be re-admitted due to pain or had to stay longer than 1 day due to the need for better pain control.

Deaths (in-hospital/30-day survival, 90-day survival, and survival until the end of the study) related to disease progression and other causes were captured.

There were no missing data in all variables captured.

The re-admissions and the morbidity were captured up to 90 days postoperatively.

### 2.3. Matching of Patients

On completion of the data collection and the initial group comparison, propensity score matching was conducted in a 1:1 matching manner with the “nearest neighbour” technique, allowing for 0.5 matching tolerance. Two groups were created: the first group included 46 cases of patients who were discharged the next day or within 24 h of surgery, and the second one included 46 cases of patients who stayed longer in the hospital post-surgery. Post-matching investigations were performed to designate the similarity of the groups.

### 2.4. Surgical and Postoperative Recovery Details

All procedures were conducted with the Intuitive Da-Vinci X^®^ Robotic platform. An assistant port was only used in a few cases according to the needs. The surgeon preferred to use 2 × 12 mm ports in order to facilitate the insertion of the staplers, allocating them in a way that 2 “right hands” are available. In all cases, no port behind the scapular line was conducted. One of the 12 mm ports (usually number 3) was routinely enlarged to retract the specimen, and the chest drain (usually 20Fr) was inserted via port No. 2 (i.e., the camera port) after moving the camera to another port. Complex anatomical lung resection requiring chest wall resection, intrapericardial approach, sleeve anastomosis, pulmonary artery plasty, etc., although totally performed robotically without conversion to thoracotomy, were excluded from the analysis, as their recovery would be anticipated to be longer.

The surgeon performed local analgesia while creating the ports and also performed multilevel paravertebral blocks using Ropivacaine 0.2% with the allowed volume according to body weight. Oral analgesia, including codeine or morphine regimes, paracetamol, and gabapentin, was also prescribed. Patients receive patient-controlled anesthesia (PCA) as needed. Both groups received identical rest postoperative care, including mobilization within hours from surgery and physiotherapy from the day of the procedure (unless there was a late finish in the afternoon due to physiotherapist availability), use of nebulizers (Salbutamol 2.5 mgs, Ipratroprioum 500 mcg, and normal saline 0.9% 5 mls) and Carbocisteine, vomiting control, laxatives, and use of incentive spirometry, following an enhanced recovery after surgery (ERAS) protocol.

### 2.5. Approval

This study was approved by the Norfolk and Norwich University Hospital as a service evaluation project.

### 2.6. Statistical Analysis

Statistical analysis was performed with IBM SPSS Statistics for Macintosh, Version 29.0. Armonk, New York, USA: IBM Corp.

All numerical data were investigated for normality of distribution with a Shapiro–Wilk’s test (normally distributed when *p* > 0.05) and Q–Q plots (acceptable figures for normality of distribution) and were presented as the mean ± standard deviation (SD). Skewed data were presented as the median (lower value–higher value). Categorical data were presented as the number of observations and percentages.

Statistical significance was determined with a Student’s t-test for normally distributed data or a Mann–Whitney test for data that were not normally distributed or lacked homogeneity of variance (Levene’s test *p* < 0.05). Chi-square and Fisher’s Exact tests were used for a comparison of categorical data. The statistical significance level was set to a *p*-value < 0.05.

There were no patients lost from follow-up due to strict cancer pathways.

Univariate binary regression analysis was conducted in order to identify predictors of ≤24 h discharge in the matched cohort. Multivariate analysis from the identified predictors was conducted next in order to identify the independent predictors of ≤24 h discharge. Confidence intervals are reported at 95%.

## 3. Results

Within the study period, overall, 202 patients fulfilled the criteria to be included in the analysis. The mean age was 68.3 ± 9.8 years, and 114 (56.4%) patients were females. From them, 49 (24.3%) patients were discharged the next day from their surgery, whereas the remainder, 153 (75.7%), stayed longer. The age, gender, number of co-morbidities, the ASA, the PS, and the FEV_1_ were different between the two groups and hence were used in the matching process (Table 1).

After the matching process, the two groups were found to be similar in all variables investigated (Table 2).

The re-admissions were similar in the two matched groups (*p* = 0.267) and involved one re-admission for a chest infection also needing reinsertion of a chest drain, one for a stroke in the next-day discharge group, chest infection in two cases, bronchopleural fistula in one needing reinsertion of a chest drain, pleural collections needing drainage in two patients, and one case of re-admission for pain in the longer-discharge group (Table 3).

Although patients who developed complications (during and after their hospitalization) were the same in number (Table 3) within the two groups (*p* = 0.126), more air leak issues were documented in the longer-discharge group (21.7% vs. 4.4%, *p* = 0.024). At the same time, the need for IV antibiotics was captured to be more frequent in that group (15.2% vs. 4.4%, *p* = 0.029) versus the next-day discharge group.

Fewer lobectomies were performed in the next-day discharge group when compared with the number of lobectomies performed in the longer-stay group (34.8% vs. 63%, respectively, *p* = 0.029). More complex sublobar resections were performed in the next-day discharge group versus the longer-stay one (47.8% vs. 26.1%, *p* = 0.023). The duration of surgery was shorter in the next-day discharge group (132.1 ± 65.9 min vs. 179.1 ± 76.6 min, *p* = 0.046), and these patients were mostly operated on in a morning (am) theater slot (71.7% vs. 32.6%, *p* = 0.018). All these are shown in Table 3.

The overall LOS was shorter in the next-day discharge group versus the longer-stay discharge group (median 1 day vs. 3, respectively, *p* < 0.001). In the longer-stay group, the LOS was weeks in some cases (Table 3).

All other variables investigated were similar between the two groups (Table 3).

Univariate analysis showed that sublobar anatomical lung resection (over lobectomy), shorter duration of operation, administration or need for IV antibiotics, more air leak issues and morning operating slots were possible predictors of a next-day discharge. A multivariate analysis of the above predictors identified sublobar anatomical lung resections, shorter durations of the procedure, and morning operating to be independent predictors of a next-day discharge (Table 4).

## 4. Discussion

The main findings of the present study are that matched patients who are discharged the next day after their anatomical lung resection do not show increased re-admission rates or other sequelae, i.e., more complications or death when compared with patients who stay longer. A sublobar anatomical lung resection, shorter duration of surgery, and morning operating were independent predictors of a next-day discharge. It can be concluded that a next-day discharge is feasible and safe, irrespective of the patient’s characteristics (i.e., age, gender, lung function tests, co-morbidities, and overall fitness) but can be augmented from surgical practice (i.e., sublobar resections, morning operating, and a short duration of surgery).

It was previously shown that patients undergoing RATS anatomical lung resections could be discharged the next day if they were of better performance status, younger age, had better lung function (i.e., higher FEV_1_ and TLCO), and others [7,8]. Therefore, fitter patients would be, by definition, expected to go home quicker and be re-admitted less. Our results show that regardless of their age, gender, co-morbidities, and fitness status, patients undergoing RATS lung resection can be safely discharged the next day without risk of increased rates of re-admissions or other issues.

The results from our study verify what has been published already and what seems logical regarding lung resections; shorter procedures, with less volume of a lung resected (i.e., sublobar anatomical lung resections) and performed in the morning provide a high probability of next-day discharges [7,8].

With regards to morning surgery, this can possibly be explained by the fact that patients have more time to recover and mobilize around the ward post-surgery, and after spending a night in the hospital, they feel more reassured to be discharged the next day. However, despite the fact that there were patients operated on in the afternoon who left the hospital the next day, it is logical to assume that late finishes in theaters will not allow patients to mobilize as early as those who have the procedure finishing earlier in the day. Hence, this could have led to delays in discharging the patients.

A similar number of complex sublobar anatomical lung resections were documented in the next-day discharge group. Hence, despite the fact that these procedures are more technically complex and demanding, they did not result in patients staying longer in the hospital.

Issues with air leaks were found to be more frequent in the group that stayed longer [10]. Although patients from both groups were discharged with flutter bags, on some occasions, this was not feasible and hence, this was a factor defining the timing of discharge. Based on this, the strategy of meticulous dissections to avoid lung lacerations and minimization of air leaks was reconsidered by our team, i.e., the use of reinforced staplers, coverage of raw surfaces with agents to reduce air leaks, and others. Air leak presence failed, though, to be an independent predictor of a next-day discharge. This factor of delayed discharge needs further investigation, with studies investigating the impact of air leak prevention measures (for example, the use of reinforced staplers in difficult fissures or emphysematous patients, for example, or the use of tissue patches, glues, etc.) in the early discharge of patients receiving RATS anatomical lung resection is warranted.

Discharging the patients on the next day, apart from being clinically beneficial if safe, could potentially provide a financial benefit, as well. According to our data, patients who stayed longer in the hospital had a median length of stay of 3 days, some of which were in a CCC-level bed (four patients). The range was also wide, from 2 to 16 days. Consequently, the above less in-hospital and less CCC stay could lead to cost benefits, but a separate analysis is warranted to prove this assumption, and hence further studies are needed in this direction [11].

All patients in our study followed an enhanced recovery after surgery (ERAS) protocol. Previous studies have shown reductions in length of stay and hospital costs for patients following an ERAS protocol for both lung resections [12,13] and robotic surgery [14]. As well as the aforementioned factors associated with a faster discharge post-RATS anatomical lung resection, a well-designed ERAS protocol is also likely to be a key contributing factor for shortening hospital stays and reducing costs. Furthermore, ERAS protocols have been associated with clinical benefits of fewer postoperative complications, reduced durations of chest drain insertion, and lower rates of chest drain reinsertion [13].

This study has limitations. Firstly, it involves one surgeon’s experience who, however, has totally switched to robotic operating. Reporting only one surgeon’s experience is important because, in our opinion, it minimizes variations in practice and the surgical trauma induced during surgery. However, this minimizes generalizability, and hence, multi-centered validation of the results is warranted. Secondly, it is a retrospective analysis and despite propensity matching, bias cannot be entirely excluded. Further studies, ideally randomized, would be necessary to prove our hypothesis. Thirdly, there was variability in the ranges in some of the variables shown to be independent predictors of next-day discharge and, more specifically, of the sublobar anatomical lung resection, and as such, this finding needs to be carefully considered when interpreting this outcome. Fourthly, the next-day discharge group included a very small number of patients who had to stay just over 24 h because of issues non-related to the discharging team, for example, delays in the dispatch of medicines, delayed transportation, etc. Lastly, the number of patients is limited, as this study is a single-institution study.

Based on all the above, we would propose teams explore next-day discharge in patients who have sublobar anatomical uncomplicated procedures in the morning and without air leaks immediately postoperatively.

## 5. Conclusions

In conclusion, a next-day discharge following a RATS anatomical lung resection can be safely achieved in patients who have uncomplicated sublobar anatomical surgery in the morning and who are independent of fitness, age, or other patient characteristics.

## Figures and Tables

**Table 1:** Demographics and clinical details for all patients N = 202 according to the timing of discharge (before propensity matching).

Variable	Next Day n = 49	Longer n = 153	*p*-Value
Gender (females) **(n, %)**	34 (69.4%)	80 (52.3%)	0.035 *
Age in years **(mean ± SD)**	66.3 ± 7.1	71.8 ± 10.3	0.041 **
PS **(median, range)**	0 (0–2)	1 (0–2)	**0.088 ^#^**
ASA **(median, range)**	2 (2–4)	3 (2–4)	**0.033 ^#^**
FEV_1_ (%) **(mean ± SD)**	76.9 ± 10.1	62.1 ± 13.2	0.021 **
TLCO (%) **(mean ± SD)**	79.1 ± 9.2	72.9 ± 11.4	0.418 **
BMI **(mean ± SD)**	28.1 ± 7.3	26.8 ± 9.7	0.516 **
No. of co-morbidities ^1^ **(median, range)**	1 (1–4)	2 (1–5)	**0.038 ^#^**

d/c: discharge, PS: performance status, FEV_1_: Forced Expiratory Volume in 1 s, TLCO: Transfer Factor for Carbon Monoxide, BMI: body mass index, SD: standard deviation, No: number, *: chi-square test, **: Student’s *t*-test, ^#^: Mann–Whitney test. ^1^ All co-morbidities are listed in the patients’ notes.

**Table 2:** Demographics and clinical details for patients N = 92 according to discharge (after propensity matching).

Variable	Next Day n = 46	Longer n = 46	*p*-Value
Gender (females) **(n, %)**	31 (67.4%)	31 (67.4%)	0.946 *
Age in years **(mean ± SD)**	68.3 ± 7.1	67.4 ± 8.3	0.929 **
PS **(median, range)**	1 (0–2)	1 (0–2)	0.823 ^#^
ASA **(median, range)**	2 (2–4)	2 (2–4)	0.833 ^#^
FEV_1_ (%) **(mean ± SD)**	69.8 ± 8.1	67.9 ± 10.2	0.899 **
TLCO (%) **(mean ± SD)**	73.2 ± 8.2	74.8 ± 9.8	0.818 **
BMI **(mean ± SD)**	27.8 ± 8.3	27.1 ± 8.2	0.687 **
No. of co-morbidities **(median, range)**	1 (1–3)	1 (1–3)	0.988 ^#^

d/c: discharge, PS: performance status, ASA: American Society of Anesthesiologists score, FEV_1_: Forced Expiratory Volume in 1 s, TLCO: Transfer Factor for Carbon Monoxide, BMI: body mass index, SD: standard deviation, No: number, *: chi-square test, **: Student’s *t*-test, ^#^: Mann–Whitney test.

**Table 3:** Operative and clinical outcomes for patients N = 92 according to discharge.

Variable	Next Day n = 46	Longer n = 46	*p*-Value
Re-admissions **(n, %)**	2 (4.3)	6 (13.0)	0.267 ^$^
Duration of surgery **(mean ± SD)**	132.1 ± 65.9	179.1 ± 76.6	0.048 **
LOS **(median, range)**	1 (1–4)	3 (2–16)	**<0.001 ^#^**
Morning session ^1^ **(n, %)**	33 (71.7)	15 (32.6)	0.018 *
Overall complications ^2^ *Respiratory* *Air leak issues* *Cardiac* *Renal* *Stroke* *IV Abx* *Intervention* *Pain related* **(n, %)**	10 (21.7) 2 (4.4) 2 (4.4) 1 (2.2) 2 (4.4) 1 (2.2) 2 (4.4) 1 (2.2) 0 (0.0)	14 (30.4) 2 (4.4) 10 (21.7) 1 (2.2) 1 (2.2) 1 (2.2) 7 (15.2) 3 (6.5) 1 (2.2)	0.126 ^$^ 0.638 ^$^ **0.024** **^$^** 0.998 ^$^ 0.877 ^$^ 0.999 ^$^ **0.029** **^$^** 0.998 ^$^ 0.998 ^$^
Type of resection Lobectomy SALR **(n, %)**	16 (34.8) 30 (65.2)	29 (63.0) 17 (37.0)	0.029 *
Complex SALR ^3^ **(n, %)**	22 (47.8)	12 (26.1)	0.023 *
Return to theater **(n, %)**	0 (0.0)	0 (0.0)	0.999 *
Conversion to open **(n, %)**	0 (0.0)	0 (0.0)	0.999 *
Preoperative CCC booked ^4^ **(n, %)**	4 (8.7)	4 (8.7)	0.998 ^$^
Escalation to CCC **(n, %)**	0 (0.0)	1 (2.2)	0.998 ^$^
Histopathology *Primary Lung Ca* *Metastasis/other Ca* **(n, %)**	28 (60.9) 10 (21.7)	36 (78.3) 7 (15.2)	0.529 *
In-hospital/30-day mortality **(n, %)**	0 (0.0)	0 (0.0)	0.999 ^$^
90-day mortality **(n, %)**	0 (0.0)	0 (0.0)	0.999 ^$^

d/c: discharge, LOS: length of stay, IV ABx: intravenous antibiotics, SALR: sublobar anatomical lung resection, CCC: Critical Care Complex, Ca: cancer, SD: standard deviation, No: number, *: chi-square test, **: Student’s *t*-test, ^#^: Mann–Whitney test, ^$^: Fisher’s exact test. ^1^ Morning session is perceived as a session finished by 12:00 midday; ^2^ number of patients who were found with 1 or more complications; ^3^ complex SALR = all sublobar resections apart from lingulectomy, S6, or left upper trisegmentectomy; ^4^ patients could have been discussed at the high-risk meeting, but instead of CCC, the patient was kept in recovery for a longer period of time and was then sent to the ward.

**Table 4:** Univariate and multivariate analyses of next-day discharge predictors N = 92 patients.

Variable	B	Exp (B)	C.I.	*p*-Value	B	Exp(B)	C.I.	*p*-Value
SALR	1.172	3.229	1.450–7.192	**0.004**	1.835	6.265	1.686–23.275	**0.006**
Shorter duration	0.012	1.019	1.001–1.022	**0.019**	0.013	1.013	1.002–1.025	**0.028**
Less air leak issues	1.810	6.111	1.258–29.693	**0.025**	1.663	5.275	0.546–50.965	0.151
Morning session	0.998	2.227	1.103–1.289	**0.009**	1.004	3.445	1.203–1.899	**0.032**
Fewer IV antibiotics	2.089	8.077	0.952–68.561	0.069				
Complex SALR	−0.201	0.818	0.192–3.479	0.786				

C.I.: confidence interval, SALR: sublobar anatomical lung resection, IV: intravenous.

## Data Availability

All data are reported within this manuscript. Raw data can be obtained on request from the corresponding author.
